# Threats to large brown algal forests in temperate seas: the overlooked role of native herbivorous fish

**DOI:** 10.1038/s41598-017-06394-7

**Published:** 2017-07-20

**Authors:** Fabrizio Gianni, Fabrizio Bartolini, Alexis Pey, Mathieu Laurent, Gustavo M. Martins, Laura Airoldi, Luisa Mangialajo

**Affiliations:** 1 Université Côte d’Azur, CNRS, ECOMERS, Nice, 06108 France; 2 Sorbonne Universités, UPMC Univ Paris 06, INSU-CNRS, UMR 7093 Laboratoire d’Océanographie de Villefranche (LOV), Villefranche sur mer, 06230 France; 3 Department of Biology, Faculty of Sciences and Technology & Centre for Ecology, Evolution and Environmental Changes/Azorean Biodiversity Group (cE3c), University of the Azores, 9501–801 Ponta Delgada, Portugal; 4 Dipartimento di Scienze Biologiche, Geologiche ed Ambientali BIGEA, University of Bologna, UO CoNISMa, Ravenna, 48123 Italy

## Abstract

Canopy-forming algae are declining globally due to multiple disturbances. This decline has recently been on the increase due to the spread of some tropical herbivorous fishes. This new phenomenon has drawn attention to the effects of fish herbivory in temperate areas, which have been assumed to be negligible compared to that of invertebrates, such as sea urchins. In this study, the impact of a Mediterranean native herbivorous fish (S*arpa salpa*, salema) was assessed on the canopy-forming seaweed *Cystoseira amentacea* var. *stricta*. *Cystoseira amentacea* forms belts in the infralittoral fringe of wave-exposed shores, which has so far been considered a refuge from fish herbivory. To test the effects of salema feeding on natural *C. amentacea* belts, an innovative herbivore deterrent device was conceived. Salema had a significant effect on *C. amentacea* by decreasing algal size, biomass and fertility, by up to 97%. The results suggest that the contribution of salema feeding to the loss of *Cystoseira* forests in the Mediterranean may have been overlooked. In addition, the analysis of temporal and spatial patterns of salema landings in the Mediterranean Sea suggests that salema abundance may have increased recently. Thus, along with invertebrate herbivory and anthropogenic stressors, fish herbivory may also represent a potential threat to algal forests in temperate areas.

## Introduction

Marine forests of large brown seaweeds are unique habitats that support a great variety of organisms in coastal zones worldwide and are comparable to terrestrial forests for the services they provide^1, 2^. Several species, mostly belonging to the orders Fucales and Laminariales, can dominate both shallow and deep-water marine ecosystems (up to their light compensation limit)^3, 4^.

Algal forests are exposed to multiple disturbances that have caused a decline in their abundance in many coastal areas of the world^5, 6^. A recent survey highlighted that, although global drivers could affect kelp forests at multiple scales, local stressors and regional variations in the effects of these drivers dominate kelp dynamics^7^. Species thriving in shallow-water zones are the most strongly affected because they are located in a boundary environment, exposed to impacts of both terrestrial and marine origin. For instance, contaminants^8^, nutrient enrichment^9^, sediment loads^10^, increase in seawater temperature^11^ and habitat alteration, resulting from coastal urbanisation^6^, are well-known factors responsible for the loss of large brown seaweeds.

Fucoids and kelps can also be highly vulnerable to herbivory^12^. Outbreaks of sea urchins, due to natural fluctuations, but more often as an indirect effect of overfishing of their predators, are responsible for the depletion of macroalgal communities and the subsequent formation of extensive barren grounds^13, 14^. This phenomenon has been observed in many coastal areas^1^ and nowadays sea urchin barren grounds are common in many temperate bioregions. As a consequence, sea urchins are considered as the primary herbivorous consumers of algal canopies in the subtidal zone of temperate areas^15^. In the intertidal zone, where sea urchins do not usually occur, gastropods and limpets are often considered as the primary herbivores^16, 17^. Therefore, in both subtidal and intertidal temperate ecosystems, herbivory is generally associated with invertebrates.

In contrast, the role of herbivorous fishes in regulating macroalgal vegetation is highly variable. In tropical areas, they play an important role in limiting shifts from coral- to macroalgae-dominated communities^18^. In temperate zones, fish grazing control is assumed to be lower or negligible in comparison to grazing from invertebrates^19–21^. The gradient of control from herbivorous fishes to invertebrates at high latitudes is hypothesized to be driven by the decrease in herbivorous fish species diversity and abundance^22^.

However, some evidence suggests that the role of herbivorous fishes in temperate regions may have been underestimated. Verlaque observed that the feeding behaviour of *Sarpa salpa* (salema), the only native herbivorous fish in the Mediterranean Sea, is particularly selective, contributing to maintaining high algal biodiversity^23^. Salema are also able to determine the distribution of some *Cystoseira* species, restricting grazer-sensitive species to spatial refuges, in either very shallow or deep areas, where fish grazing pressure is lower^24^. The same observations were reported from central Portugal, where *S. salpa* is responsible for reducing kelp biomass and restricting the survival of recruits to crevices^25^. Taylor and Schiel did analogous observations on *Odax pullus* in New Zealand, which is able to significantly reduce the cover and biomass of the kelp *Durvillaea antarctica* ^26^. In addition, recent studies have highlighted the impact of tropical herbivorous fishes, by expanding their distribution range into temperate areas, on canopy algae^27^.

The aim of the present study was to quantify the potential impact of *Sarpa salpa* on large brown algal forests and to provide evidence of a potential increase of salema over the last decades. The herbivory effect of *S. salpa* was investigated on size, biomass and fertility of *Cystoseira amentacea* var. *stricta* (hereafter *C. amentacea*), a species forming narrow belts in the very shallow infralittoral fringe of the Mediterranean Sea, which is considered a refuge from fish herbivory^24, 28^. In order to experimentally manipulate fish grazing on *C. amentacea*, an innovative herbivorous fish deterrent device was used (DeFish – herbivorous Fish Deterrent for the conservation and restoration of algal forests (Gianni *et al*. in prep.)). The hypotheses of the present study were: i) herbivore exclusion devices are able to effectively reduce *S. salpa* feeding on *C. amentacea*, ii) *C. amentacea* size, biomass and fertility are lower due to fish herbivory if *S. salpa* is not excluded, iii) the magnitude of these effects is greater in low compared to upper level zones of the infralittoral fringe, that are assumed to be less accessible to fish.

To assess potential recent increases of *S. salpa* abundance, temporal patterns in fish landings of salema within the different basins of the Mediterranean Sea were analysed.

## Materials and Methods

### Study area and species

**Figure 1:**
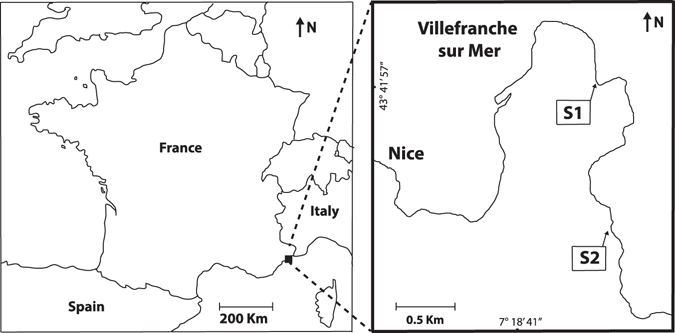
Study area. The two sampling sites are located in Villefranche Bay, French Riviera, NW Mediterranean Sea. Maps were made by using Adobe^®^ Illustrator^®^ software CS6.

**Figure 2:**
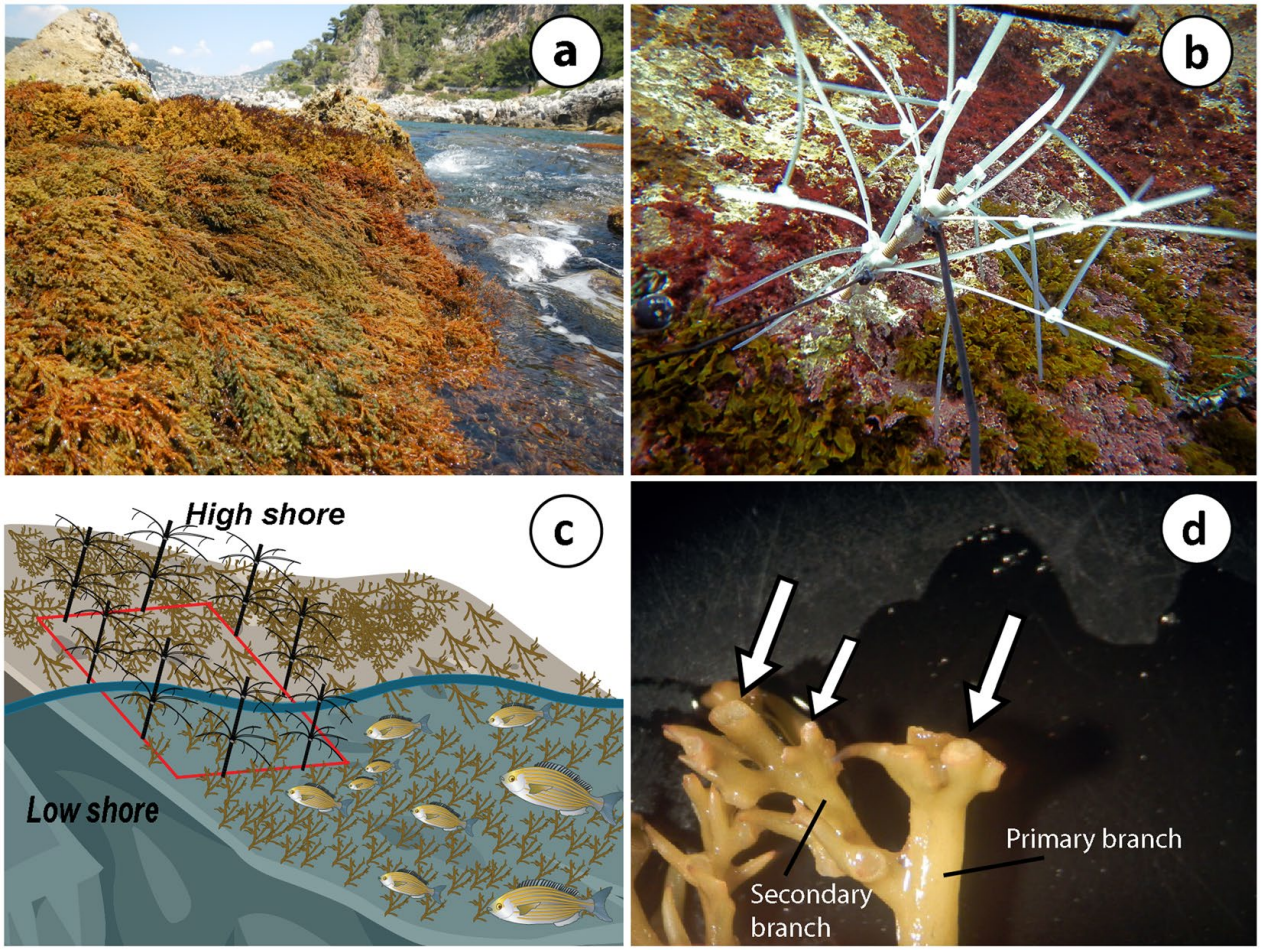
(**a**) *Cystoseira amentacea* belts in the infralittoral fringe; (**b**) the deterrent device DeFish used to deter fish grazing on *C. amentacea*; (**c**) schematic representation of the two vertical zones of the infralittoral fringe characterized by long and short *C. amentacea* branches; a protected plot with DeFish is also shown on the shore; (**d**) detail of the primary and secondary *C. amentacea* branches with fish bites (white arrows). Photos by Bartolini F. Figure 2c attributions: salema and *Cystoseira* illustrations (Tracey Saxby, Integration and Application Network, University of Maryland Center for Environmental Science (ian.umces.edu/imagelibrary/)).

The experiment was performed between March and June 2014 at two randomly chosen sites in Villefranche Bay, French Riviera (43°41′32″N, 7°18′58″E; Fig. 1). Both sites were characterized by dense intertidal fringing belt populations of *Cystoseira amentacea* (Fig. 2a).

*C. amentacea* is a habitat-forming species, common along North-Western Mediterranean shores^29, 30^, where it supports complex food webs on rocky-bottoms^31, 32^. Its threatened status is recognised by the Bern and Barcelona Conventions. *C. amentacea* is a long-lived species with a seasonal pattern of growth between March and July. The base is sympodial, formed by a creeping axis from which multiple axes grow. Branches can reach 40 cm length and are lost in late summer, when the dormant season begins. Abundant receptacles, from a few millimetres to 2 cm long, begin to develop at the apical part of all branches at the end of April^33^. Since zygotes are heavy and tend to sink close to the parent individual^34^, *C. amentacea* is characterised by a very limited dispersal ability (dozens of centimetres according to Mangialajo *et al*.^35^, even if rare events of distant dispersal were suggested by recent studies^29, 36^).

### Experimental design and set up

An innovative herbivore deterrent system (DeFish) consisting of a 20 cm long plastic threaded rod with three groups of five cable ties (18 cm long) was designed to deter fish grazing. Each group of cable ties was attached with silicon glue at different heights on the rod and kept straight with a plastic bolt screwed onto the rod (Fig. 2b). To deter fish from passing through the devices, smaller cable ties (10 cm long) were attached perpendicular to the longer ties. Rods were then screwed to drop-in anchors fixed inside holes (2 cm depth) that were drilled into the rock along each side of the protected plots.

Compared to the cages that are often used in herbivore exclusion experiments^12^, these devices do not require maintenance or cleaning, do not affect light penetration and can be easily removed. However, their installation is time-consuming, logistically challenging for very shallow positions on the shore, and can have a relatively high impact on these fragile environments (involving drilling the rock and fixing by epoxy putty). Therefore a split-plot design^37^ was applied, at only two sites, to optimise the experimental robustness, while keeping the impact on the forests and logistical constraints at minimum.

Twelve plots (the blocking factor, 40 × 40 cm) were randomly set up in the infralittoral fringe at each of the two sites, spaced several meters apart. Following the split-plot design, two subplots (high-zone and low-zone subplots) were attributed to each plot. The high zone subplot corresponds to the upper level of the infralittoral fringe, where belts of *Cystoseira amentacea* are mostly emerged due to wave and tide action and therefore are expected to be less grazed. The low-zone subplot corresponds to the lower level of the infralittoral fringe, where *C. amentacea* belts are almost always immersed and therefore more accessible to fish (Fig. 2c). Then, each plot was randomly assigned to one of three treatments: protected, un-manipulated control and artefact control, for a total of 4 replicated plots per treatment and per site. Protected plots were completely enclosed using at least six deterrent devices fixed on the edges of the plots. In the artefact control plots, three small devices (10 cm high), made with the same materials, were fixed on the edges of the plots, so as to check for a possible effect of the manipulation on *C. amentacea*, and, at the same time, allow fish access. Non-manipulated control plots were marked with epoxy putty.

### Data collection

The experiment started at the beginning of the *Cystoseira amentacea* growth period, in March 2014. Fish herbivory was estimated by assessing the number of bites on *C. amentacea* branches. *Sarpa salpa* bite marks are evident since the algal branch is clean cut (Fig. 2d). Crabs can also clean cut the branches, but their action is sporadic and limited compared to salema grazing (Gianni *et al*. under review). Bites cannot be confused with natural broken branches since these are frayed and not precisely broken. The sites selected were virtually inaccessible to people, so that trampling effects could not affect *Cystoseira* branches. A negative effect of the devices on *Cystoseira* branches can be also excluded since they did not swing too much with wave action. To assess the effect of fish feeding on *C. amentacea*, algal size (maximum length in cm), biomass (wet weight in g) and fertility (number of receptacles) were also estimated. Identification of single *C. amentacea* individuals is difficult, because of its sympodial bearing. Thus, all measurements were completed within a 12.5 cm^2^ reference surface (4 cm diameter circle), assumed to be a proxy of an individual, at the centre of both the high and the low zone of every plot. This approach can mitigate potential biases associated with the split-plot design (i.e. a reduced spatial independence of data). The number of bites was visually assessed in the field together with algal size that was measured with a ruler (accuracy 0.1 cm) in March (before installing the devices), May and June. At the end of June, the experiment was concluded to avoid *C. amentacea* individuals starting to lose their fronds due to high summer temperatures. Therefore, all *C. amentacea* individuals inside the reference surfaces were harvested and transported to the laboratory to estimate biomass and the number of receptacles. The destructive sampling was performed only once, at the end of the experiment, in order to avoid damaging the plots.

Density and size of *Sarpa salpa* were estimated twice, in March and June, during the experiment, using visual counts along ten replicated transects (25 × 5 m) in the experimental area. Transects were performed parallel and perpendicular to the coast on a rocky-sea bottom (maximum depth: 3 m)^38^. Salema biomass was calculated by using the length-weight relationship reported on FishBase (http://www.fishbase.org).

The datasets analysed during the current study are available from the corresponding author on request.

### Statistical analyses

Differences in response variables among the treatments and zones were tested by using permutational multivariate analysis of variance (PERMANOVA)^39^. The use of a multivariate approach on univariate data is justified if a permutational analysis is performed based on Euclidean distance matrices of the measured biological variables, as specified in Anderson *et al*.^37^. In order to test the homogeneity of dispersions, PERMDISP tests were performed, before permutational analyses, on medians and tables to fit with univariate data^37^. All PERMDISP tests are reported in the Supplementary Materials. The full split-plot model analysed with PERMANOVA was composed by the fixed factors Treatment with three levels (protect, control, artefact control), Zone with two levels (High and Low Zone) and by the random factor Plot (n = 4), nested in Treatment. The analyses were run using ‘Type I Sum of Square’, so that the terms were fitted sequentially, according to the split-plot design^37^. Separate analyses were performed for each time to avoid temporal dependence of data. The sampling sites were also analysed separately because at site 2 a storm in May partially damaged the experimental setup and the deterrent devices did not manage to exclude fish completely. The two sites are therefore referred to hereafter with their local names (i.e. S1: Pointe du Rubé, S2: Pointe de la Cuisse). PERMANOVA p-values were obtained based on 9999 permutations of residuals under the reduced model. Statistical significance was set at the conventional *p* < 0.05 level, except when dispersion tests were significant and, in this case, a more conservative *p*-value (*p* < 0.01) was considered. Pairwise tests were performed on factors or interactions giving statistically significant results, calculating Monte Carlo p values in the case of a low number of possible permutations ( < 100). Analyses were completed with the Primer 6 & PERMANOVA + software package.

The net growth potential and reproductive potential of *Cystoseira amentacea* were estimated at both sites. The net growth potential was calculated as the difference in algal branch length between June and March, whereas the reproductive potential was a standardisation in relation to the maximum fertility (number of receptacles multiplied by 100 and divided by the highest number of receptacles recorded at each site). The effect size for protection and zone was calculated on these variables using the log-response ratios^40^ for each zone in each treatment as:

1 $${R}_{t}=\,\mathrm{ln}(\frac{{\bar{x}}_{t,z}}{{\bar{x}}_{c,z}})$$

where R_t_ is the log-response ratio for the treatment *t*, and ${\overline{x}}_{t,z}$ and ${\overline{x}}_{c,z}$ are the mean values of net growth potential and reproductive potential, respectively calculated for the treatments protection or artefact control (*t*) and the treatment control (*c*), in each zone (*z* = High or Low).

### Potential patterns of change in salema abundance

Potential patterns of temporal and spatial variation in salema abundance were investigated in the Mediterranean Sea, using the Mediterranean and the Black Sea capture production quantity (1970–2014) dataset accessed via the FishStatJ (Food and Agriculture Organization, FAO). In accordance with the FAO dataset, the Mediterranean Sea was divided into seven basins (Adriatic Sea, Sardinia Sea, Gulf of Lion, Aegean Sea, Balearic Sea, Ionian Sea, Levant Sea). In order to identify a potential time pattern of the catches (in tonnes), linear trend models were performed for each basin.

## Results

### *Sarpa salpa* density and size

**Figure 3:**
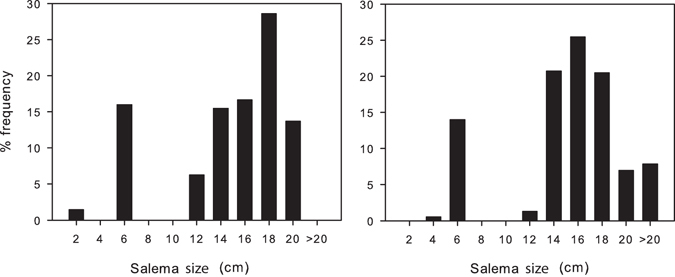
Size class distributions of *Sarpa salpa*. Percentage frequency of salema individuals per size recorded in the two visual census performed during the experiment.

The recorded *Sarpa salpa* mean abundance was 0.2 individuals/m^2^ ± 0.06 (SE), corresponding to a mean biomass of 9.88 g/m^2^ ± 2.14. Most individuals (60%) were >14 cm in length (Fig. 3).

### Fish herbivory

**Figure 4:**
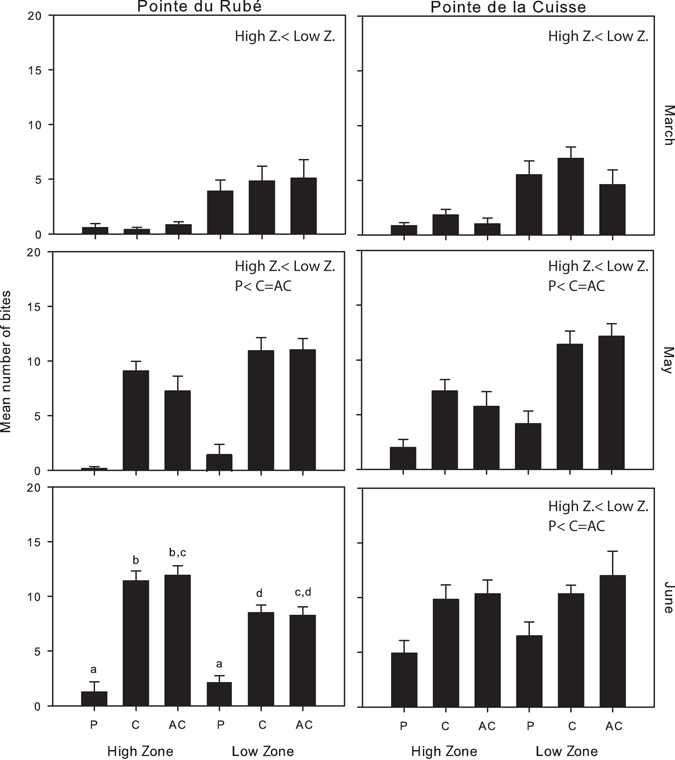
Fish herbivory. Number of fish bites/12.5 cm^2^ (mean plus SE calculated on all plots, n = 4) for each zone (High and Low) and treatment in the different months and at both sites. P: protected; C: control plots; AC: artefact control. The results of the pairwise tests on the factors Treatment and Zone are reported above the graph (see Table 1 and Supplementary Tables S1.1–2 for more details). Letters above the bars in June at Cap du Rubé indicate significant differences of the pairwise-tests on the interaction TrxZo (Table 1 and Supplementary Table S.1.1 for more details).

The number of bites recorded on *C. amentacea* ranged between 0/12.5 cm^2^ (in the High Zones of Pointe du Rubé in June) and 27/12.5 cm^2^ (in the Low Zone of an unprotected plot at Pointe de la Cuisse in June). Before setting up the deterrent devices, in March, the number of bites was already significantly greater in the Low Zone compared to the High Zone: on average 0.9 bites ± 0.3 (SE) were recorded in the High Zone and 5.1 ± 1.3 bites in the Low Zone at both sites (Fig. 4 and Table 1). Two months after the setting up of the experiment, the number of fish bites was significantly greater in unprotected than in protected plots (Fig. 4, Table 1 and pairwise tests: Supplementary Tables S1.1–2). The number of bites increased in particular in the High Zone of unprotected plots, even if this number was always lower in comparison to that recorded in the Low Zone (Fig. 4). The experimental protection in the treatment plots effectively limited fish bites at both shore levels and sites (Fig. 4). In June, a significant interaction among the factors Treatment and Zone was recorded at Pointe du Rubé, showing that the two Zones in the protected plots were not statistically different in terms of number of bites due to the effect of protection (Table 1 and pairwise tests: Supplementary Table S1.1). The interaction was not statistically significant at Pointe de la Cuisse, neither the factor Zone (Table 1), potentially due to the fact that the experimental setup was slightly damaged by storms, which may have reduced the ability of the deterrent devices to exclude fish completely.

**Table 1:** Tables of PERMANOVA calculated on the different parameters: fish herbivory (as number of bites/12.5 cm^2^), algal size (as mean maximum length in cm), biomass (in g/12.5 cm^2^) and fertility (as number of receptacles per 12.5 cm^2^), for both sites and in the different months. Tr: Treatment, Zo: Zone, Pl: plots. Statistically significant values are in bold type. ﻿Asterisks indicate that PERMDISP tests were significant (see Supplementary materials).

	df	Source	March						May						June					
			Rubé			Cuisse			Rubé			Cuisse			Rubé			Cuisse		
			MS	Pseudo-F	P(perm)	MS	Pseudo-F	P(perm)	MS	Pseudo-F	P(perm)	MS	Pseudo-F	P(perm)	MS	Pseudo-F	P(perm)	MS	Pseudo-F	P(perm)
Fish herbivory	2	**Tr**	1.00	0.21	0.82	6.25	1.94	0.17	204.7	54.12	**0.005**	97.56	14.1	**0.006**	184.29	59.65	**0.004**	66.81	6.73	**0.013**
	1	**Zo**	96.00	28.51	0.0003*	117.04	27.89	0.0009*	31.89	7.25	**0.02**	109.8	33.4	**0.0002**	21.40	9.81	**0.01**	9.37	1.52	0.25
	9	**Pl(Tr)**	4.74	1.40	0.29*	3.20	0.76	0.64	3.78	0.86	0.59	6.91	2.1	0.14	3.08	1.41	0.30	9.92	1.61	0.23
	2	**TrxZo**	0.68	0.20	0.81	1.62	0.38	0.67	3.25	0.74	0.54	9.03	2.75	0.12	12.08	5.53	**0.02**	0.84	0.13	0.87
Algal size	2	**Tr**	1.51	1.68	0.23	0.87	0.58	0.57	154.17	57.38	**7E-04**	54.66	4.7	**0.04**	162.21	65.86	**0.003**	100.2	21.29	**0.005**
	1	**Zo**	32.59	42.18	**0.0004**	135.3	67.5	0.0001*	57.6	25.46	**0.001**	276	28.3	**0.0004**	32.1	29.37	**0.0007**	207.59	95.19	**0.0001**
	9	**Pl(Tr)**	0.89	1.16	0.41	1.50	0.75	0.66	2.68	1.18	0.39	11.62	1.19	0.40	2.46	2.25	0.12	4.70	2.15	0.12
	2	**TrxZo**	0.79	1.02	0.38	0.21	0.10	0.90	4.77	2.10	0.17	26.54	2.72	0.11	0.63	0.58	0.57	0.91	0.41	0.65
Biomass	2	**Tr**													1.04E + 07	25.63	0.005*	2.11E + 07	13.71	**0.002**
	1	**Zo**													3.77E + 06	32.18	**0.0008**	3.37E + 07	9.85	**0.01**
	9	**Pl(Tr)**													4.05E + 05	3.45	**0.008**	1.54E + 06	0.44	0.87
	2	**TrxZo**													1.85E + 06	15.79	**0.001**	4.85E + 06	1.41	0.29
Fertility	2	**Tr**													44779	8.89	0.004*	66686	40.3	0.006*
	1	**Zo**													20817	8.18	**0.01**	26611	23.18	**0.001**
	9	**Pl(Tr)**													5031	1.97	0.12	1654	1.44	0.26
	2	**TrxZo**													18115	7.11	**0.01**	12901	11.23	**0.003**

### Algal size

**Figure 5:**
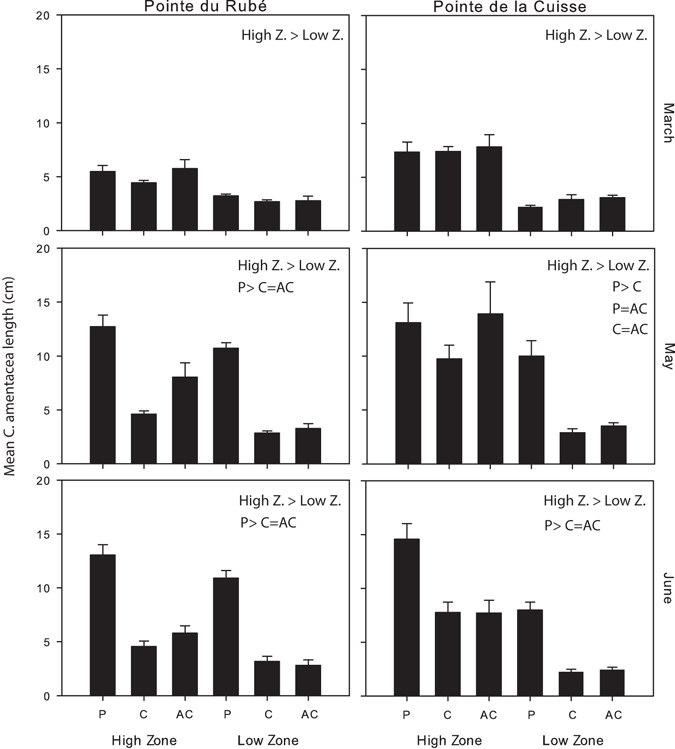
Algal size. Algal length (mean plus SE calculated on all plots, n = 4) for each zone (High and Low) and treatment in the different months and at both sites. P: protected; C: control plots; AC: artefact control. The results of the pairwise tests on the factors Treatment and Zone are reported above the graph (see Table 1 and Supplementary Tables S2.1–2 for more details).

The mean maximum length of *Cystoseira amentacea* ranged from 0.5 cm in the Low Zone at both sites in March to 27 cm in the High Zone of a protected plot at Pointe de la Cuisse in May. In March, at the beginning of the growth season, branches of *C. amentacea* were already significantly longer in the High than in the Low Zone at both sites (Fig. 5 and Table 1). After two months (and in particular in June), branches of *C. amentacea* were fully developed and significantly longer in the protected plots compared to most unprotected ones and in the High Zone respect to the Low Zone (Fig. 5; Table 1; Supplementary Tables S2.1–2). The length of branches in Control and Artefact Control plots did not differ significantly (Fig. 5 and pairwise tests: Supplementary Tables S2.1–2) and no significant interaction between the main factors was detected (Table 1).

**Figure 6:**
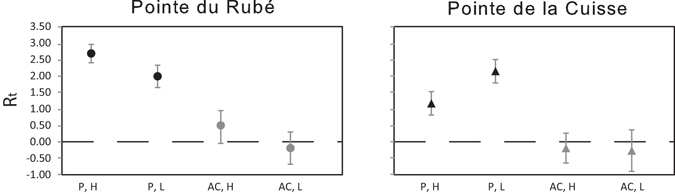
Effect sizes of protection and zones based on the net growth potential. Protection (P: protected, AC: artefact control), Zones (H: High, L: Low). Effect sizes are significant if confidence intervals do not overlap zero. R_t_: log-response ratio for each treatment.

The net growth potential of *C. amentacea*, calculated as the difference in algal length between the beginning and the end of the experiment, responded significantly and positively to herbivore protection in the two zones and sites (Fig. 6). Interestingly, similar effect sizes of the protection against herbivory on *C. amentacea* growth were recorded at the two shore levels at both sites.

### Biomass

**Figure 7:**
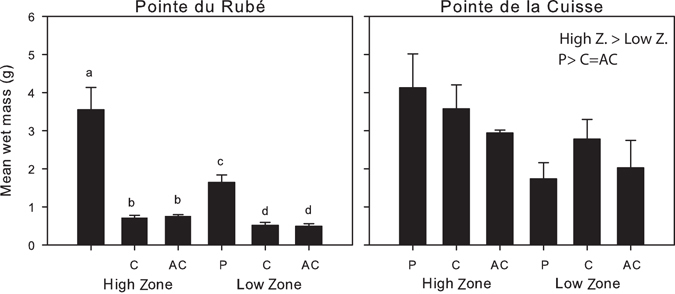
Biomass. Wet weight (g)/12.5 cm^2^ (mean plus SE calculated on all plots, n = 4) for each Zone (High and Low) and Treatment in June, at the two sites. P: protected; C: control plots; AC: artefact control. Letters above the bars indicate significant differences of the pairwise-test performed on the interaction TrxZo at Pointe du Rubé (see Table 1 and Supplementary Table S3.1 for more details). At Pointe de la Cuisse, the results of the pairwise tests on the factors Treatment and Zone are reported above the graph (see Table 1 and Supplementary Table S3.2 for more details).

The biomass, expressed as wet weight of *Cystoseira amentacea*, ranged from 0.14 g/12.5 cm^2^ in the Low Zone of an unprotected plot to almost 59 g/12.5 cm^2^ in the High Zone of a protected plot at Pointe du Rubé. A significant Treatment × Zone interaction was highlighted by the analysis of variance, but only at Pointe du Rubé (Table 1). Pairwise tests showed a greater biomass in protected compared to unprotected plots and a consistently higher biomass in the High Zone compared to the Low Zone. The High Zone was statistically different from the Low Zone also in the protected plots, but this could be due to the fact that the dispersion test was significant (Fig. 7, Supplementary Table S3.1 and PERMDISP tests). At Pointe de la Cuisse, Treatment and Zone factors were statistically significant (Table 1), showing greater biomass in protected plots and in the High Zone (Fig. 7 and Supplementary Table S3.2). Overall, the fish grazing caused up to 86% of biomass loss, particularly in the Low Zone of the Control plots (Fig. 7).

### Fertility

**Figure 8:**
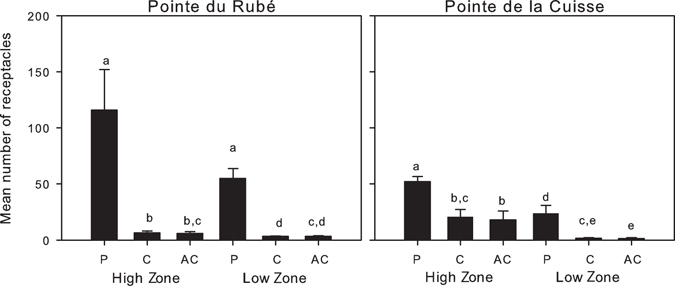
Fertility. Number of receptacles/12.5 cm^2^ (mean plus SE calculated on all plots, n = 4) for each zone and treatment in June. P: protected; C: control plots; AC: artefact control. Letters above the bars indicate significant differences of the pairwise-tests on the interaction TrxZo (see Table 1 and Supplementary Tables S4.1–2).

The number of receptacles ranged from 0/12.5 cm^2^ in unprotected plots at both sites to 3544/12.5 cm^2^ in the High Zone of a protected plot at Pointe du Rubé. *Cystoseira amentacea* individuals in protected plots and in the High Zone had a greater number of reproductive structures compared to individuals in unprotected plots and in the Low Zone (Fig. 8). In addition, *C. amentacea* individuals in the unprotected plots were often devoid of reproductive structures. The estimated loss of reproductive structures was up to 97%. The analyses of variance showed a significant Treatment × Zone interaction at both sites (Table 1). Pairwise tests showed that the number of receptacles in protected plots were significantly greater than in unprotected ones in both zones and sites (Fig. 8 and Supplementary Tables S4.1–2). However, the differences showed by the pairwise tests on this interaction have to be taken with caution, because of a significant dispersion of data in the PERMDISP analysis (Supplementary materials).

**Figure 9:**
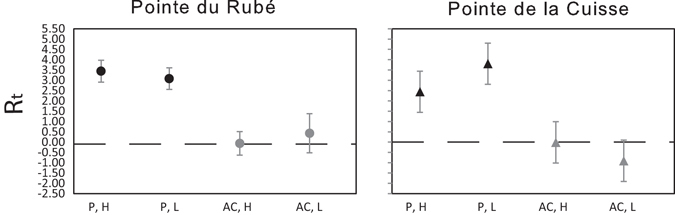
Effect sizes of protection and zones based on the reproductive potential. Protection (P: protected, AC: artefact control), Zones (H: High, L: Low). R_t_: log-response ratio for each treatment.

The reproductive potential, expressed as the ratio of the number of receptacles in each Treatment and Zone compared to the highest number of receptacles recorded at each site, confirmed a clear effect of protection in both zones of the two sites (Fig. 9). Interestingly, at Pointe du Rubé the effect of protection had similar effect sizes in both the High and Low Zones, while at Pointe de la Cuisse effect size of protection was slightly greater in the lower zone, more exposed to grazing.

### Evidence of salema increase

**Figure 10:**
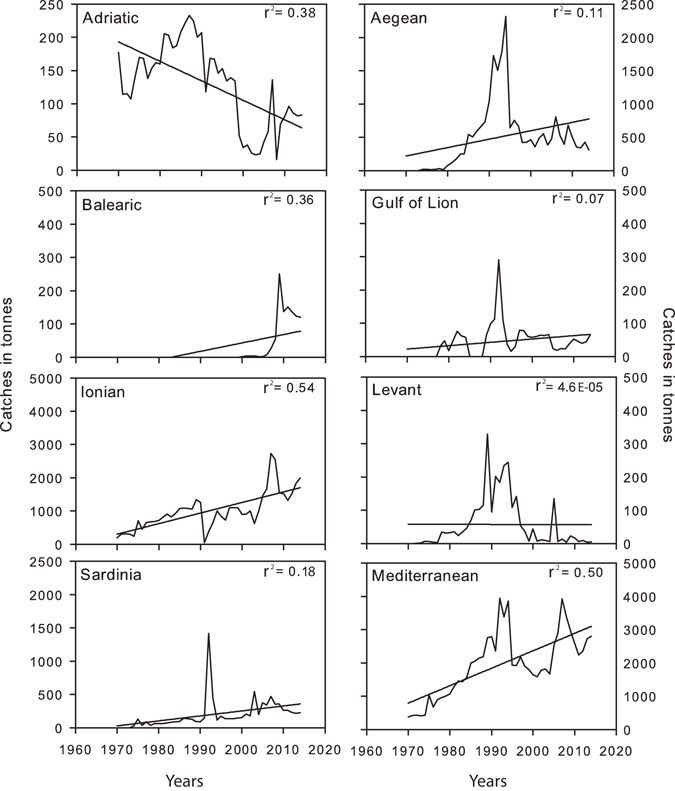
Patterns of temporal variation in salema landings. Salema catches (in tonnes) are represented for 7 distinct basins (FAO sub-areas) and the Mediterranean as a whole from 1970 to 2014﻿ (*x*-axes). Trend lines and the r-squared values are reported on each graph. Note different scales among catch axes.

Salema landings increased since 1970 in four of the seven FAO’s Mediterranean sub-areas (Aegean Sea, Balearic Sea, Ionian Sea and Sardinia Sea) and also in the Gulf of Lion (Fig. 10). In contrast, landings showed a decreasing trend in the Adriatic Sea. In the Levant Sea, salema landings showed a high fluctuation over time (Fig. 10). Despite the spatial variation among FAO sub-areas, overall and across the entire Mediterranean, landings of salema tended to increase with time, showing a 7-fold increase between 1970 and 2014 (Fig. 10). The area investigated in the present study, Villefranche Bay, lies approximately at the intersection between two FAO sub-areas (the Gulf of Lion and the Sardinian Sea, which includes both the Ligurian and the Tyrrhenian Seas). When data from these two sub-areas were combined, there was also an increasing trend in landings of salema over time. The linear trend models showed significant *p*-values for most of the basins, confirming an increase of salema catches with time: Adriatic Sea (n = 44, *F* = 26.5, *p* = 6.26E-06), Aegean Sea (n = 44, *F* = 5.23, *p* = 0.02), Balearic Sea (n = 44, *F* = 24.5, *p* = 1.18E-05), Ionian Sea (n = 44, *F* = 51.04, *p* = 7.94E-09), Sardinia Sea (n = 44, *F* = 9.9, *p* = 0.003) and Mediterranean Sea (n = 44, *F* = 44.3, *p* = 4.07E-08). *P*-values were not significant for the Levant Sea (n = 44, *F* = 0.0019, *p* = 0.96) and the Gulf of Lion (n = 44, *F* = 3.31, *p* = 0.07).

## Discussion

Marine communities are strongly regulated by top-down forces^41^. High herbivory rates can lead to regime shifts with a collapse in primary production, biodiversity and ecosystem functioning^42^. The depletion of forests of large brown seaweed and the subsequent creation of extensive barren grounds is typically mediated by sea urchins that are considered the most effective herbivores in temperate regions^15^.

The results presented in this study not only confirm that *Sarpa salpa* is able to graze up to the very shallow infralittoral fringe^24^, but also provide evidence that fish herbivory can be higher than assumed in this zone, considered to be poorly accessible to fish^24^ and mainly driven by bottom-up processes^29, 30^. Very high level of herbivory (number of fish bites) on *Cystoseira amentacea* branches was recorded in unprotected plots, resulting in a subsequent reduction in algal features. In fact, during the maximum growth period of *C. amentacea*, from March to June, grazing by salema caused up to 78% reduction in algal size (branch length). Most significantly, fish herbivory affected the biomass of *C. amentacea* up to 86% and fertility up to 97% in the unprotected plots. Interestingly, it was observed that *C. amentacea* branches lacking receptacles seemed to be less intensively grazed than branches bearing reproductive structures, probably because they are rich in fatty acids^43^. No epiphytes were present on *C. amentacea* branches during the experiment, so they could not have influenced the feeding behaviour of salema^44^.

Differences in grazing along the vertical gradient were observed in the present study, probably because the high level on the shore is accessible to fish only during high tides and with high wave action. Some previous works hypothesised that the physical environment, and in particular the rhythm of emersion/immersion by tides and waves, plays a major role in the development of *Cystoseira* shallow belts in the Mediterranean Sea^45^. Other studies suggest that grazing is a paramount factor affecting the vertical distribution, and in particular the lower limit, of such assemblages^24, 35^. The present study, based on deterrent devices that were unable to exclude fish completely, did not allow to properly test this hypothesis, as their herbivory-controlling effect may have been dependent on the level of the shore. However, it is worth noting that differences in herbivory and *C. amentacea* features between High and Low Zones were reduced due to the fish deterrent devices, becoming non-significant for the number of bites and the number of receptacles.

The temporal variation recorded in the study was a consequence of the seasonal growth of *C. amentacea* primary branches. Interestingly, at the very beginning of the experiment, when *C. amentacea* biomass was low, herbivory was already very high at the Low Zone, with a particularly clear impact on *C. amentacea* size.

Evidence of the very important role of native herbivorous fishes in temperate areas was also obtained in the South West Pacific. In this region, labrids can remove the entire primary lamina of adult kelp, causing extensive biomass loss and possibly having a significant impact on nutrient cycling^26, 46^. Kelp recruits and juveniles may escape fish herbivory only at wave-exposed sites and under dense canopies that help reduce grazing pressure^26^. A similar phenomenon has been observed in central Chile, where the blenny *Scartichthys viridis* seems to be able to regulate the presence of a large number of foliose macroalgae in the intertidal zone^47^.

In contrast to the results presented here, Vergés *et al*. recorded a much lower loss of *C. amentacea* biomass due to *S. salpa* feeding in the Balearic Islands (10% compared to an average of 85% in the present study)^24^. The recorded difference can be explained by a five times greater density of herbivorous fish at the studied site (0.2 ind./m^2^ ± 0.06, mean ± SE) than at the Balearic Islands site (0.04 ind./m^2^). In fact, fish density measured in Villefranche Bay is among the highest values reported from the Mediterranean^48–51^. Unfortunately, historical data on salema abundance, useful to assess their potential increase, are not available at this site, to our knowledge.

Natural and human-driven fluctuations of marine organism densities are common, but can often go unnoticed especially for species that are not targeted by fisheries, such as salema. FAO data, even if they need to be interpreted with caution, as they are not standardized regarding fishing effort, provide evidence of a potential increase in the abundance of salema in the Mediterranean since 1970. Increasing salema density over time was also recorded in the Portofino tuna nets (Ligurian Sea, Italy) from the 1950s to the 1970s (data to be taken with caution due to differences in fishing effort/gear^52^) and in Portuguese waters in the 1980s^53^. However, targeted long-time series are scarce, and it is difficult to assess the exact scale and extent of this potential phenomenon. These possible increases may be linked to several factors, most likely in interaction, such as global warming, changes in fishing pressure and decline of predators.

Global warming is driving the rise in seawater temperature^54^, often associated with the spread of invasive species or the proliferation of native species^27, 55^. Higher temperatures accelerate metabolic rates^56^ and may favour herbivorous fishes. A striking example is Tosa Bay in southern Japan, where the recent rise in water temperatures enhanced the grazing rate of some tropical fishes, already present in that area, and triggered a shift from kelps to corals^57^. Also in the Mediterranean Sea, the sea surface temperature is rising and this trend is expected to continue to the end of this century^58^; consequently the thermal habitat suitability of salema may increase^59^.

Changes in fishing pressure can favour an increase of *S. salpa* abundance, as it h﻿as already been recor﻿dedwithin s﻿ome ﻿Ma﻿rine Protected Areas (MPAs)^50, 60–62^, probably due to fishing regulations. It is worth noting that an increase in salema density, following protection, has been claimed as one of the potential causes of the depletion of large brown seaweed forests in the late nineties in the Portofino MPA^63^.

Although salema is traditionally fished in several areas, it has never had a high commercial value at fish markets, and data on its abundance are not commonly recorded. Discussions with fishermen in the French Riviera highlighted that the declining demand for Mediterranean fishes, due to the more widespread market distribution of species from more productive environments such as the North West Atlantic, may have caused a corresponding reduction in catches of less valuable species, including salema. At present, at the study area (French Riviera), *S. salpa* is mostly caught accidentally with non-selective gear. The same considerations may apply to other regions, but a coordinated collection of data would be necessary to estimate a possible decrease in herbivorous fishes catches in recent decades.

Another important driver that may have increased salema abundance is the depletion of their predators, such as shark, grouper and leerfish (according to FishBase), due to overfishing^64, 65^, but relevant data are scarce on this topic, and it is not possible to confirm this cascade effect.

In addition to fluctuations in abundance of native temperate herbivorous fishes due to these factors, several tropical herbivorous fishes have extended their ranges into temperate waters, due to climate change and other anthropogenic factors (e.g. human-facilitated introductions), with a major impact on the structure of benthic assemblages^27, 66^. Phase-shifts from large brown macroalgae dominated habitats to less productive barrens were also observed in the Eastern Mediterranean Sea when functionally diverse tropical fishes (*Siganus luridus* and *Siganus rivulatus*) invaded from the Red Sea^67^, replacing the native *S. salpa*, that sharply declined. Even if the North-Western Mediterranean Sea is not currently concerned by this phenomenon, a few individuals of *Siganus* have already been recorded in this basin^30, 68^. With the expected increase in sea-water temperature, their potential future spread in the Western Mediterranean Sea over the next decades may further threaten the *Cystoseira* forests.

In conclusion, the results of the present study highlighted the role of *S. salpa* in affecting *Cystoseira* forests and a potential increase in salema populations. Recent studies also suggested a potential role of herbivorous fish on the subtidal *Cystoseira* forests^24, 48, 69^, as well as on *Posidonia oceanica* meadows^70^, at depths where sea urchins have been generally considered as the main herbivores^14^. The present results support these observations by providing experimental evidence of the role of salema in shaping very shallow *Cystoseira* forests. In the light of these results, we argue that the effects of herbivorous fish on *Cystoseira* forests have been overlooked so far and we cannot exclude that they may have played a major role in the recent loss of algal forests recorded across the Mediterranean Sea, potentially interacting with other well-known causes of loss^5, 30, 71–73^. This is all the more true given that *S. salpa* seems to preferentially graze on reproductive structures, located in the apical part of *Cystoseira* branches. Since most *Cystoseira* species are characterised by limited dispersal ability^35, 36^, the decrease in fertility may accelerate the loss of forests. The general decrease in complexity of macroalgal communities, from large brown algal forests to turfs of filamentous or coralline algae in other regions^74^, may also be directly or indirectly linked to overlooked fluctuations in abundance of native herbivorous fishes. The present results provide important insights for the conservation of large brown algae forests, not only in the Mediterranean Sea, but also in other geographical areas potentially affected by the same unnoticed phenomenon. The conservation of marine vegetated habitats should take into consideration the role of herbivorous fishes and the assessment of their densities in space and time. The regulation of large herbivorous fish densities should be considered, especially where their abundance is high enough to represent a threat to large brown algae forests conservation or ecological restoration^75^. Devices similar to those proposed in this study, but preferably made of biodegradable materials and using less invasive procedures (without drilling rocks), may represent a solution to protect recently restored or isolated patches of algal forests and promote reproduction and recruitment. A potentially more sustainable measure would be the local regulation of salema populations involving the fishing community and enhancing the species’ commercial value.

## Electronic supplementary material

Supplementary Material
