# Supplementary material for: Threats to large brown algal forests in temperate seas: the overlooked role of native herbivorous fish
Source: Sci Rep. 2017 Jul 20;7:6012. doi: 10.1038/s41598-017-06394-7 (PMC5519706; doi:10.1038/s41598-017-06394-7)
Supplement: Supplementary file 1 — Supplementary Material [file 41598_2017_6394_MOESM1_ESM.pdf]

Threats to large brown algal forests in temperate seas: the overlooked role of native herbivorous fish

Fabrizio Gianni, Fabrizio Bartolini, Alexis Pey, Mathieu Laurent, Gustavo M. Martins, Laura Airoldi, Luisa Mangialajo

Table S1.1. Pairwise tests on fish herbivory for Pointe du Rubé. The pairwise tests were performed on the interaction, when it was significant, or on the factor 'Treatment' and 'Zone'. Tr: Treatment, Zo: Zone, H: High Zone, L: Low Zone, P: protected, C: control, AC: artefact control treatment.

March					May				
**Factor**	**Groups**	**t**	**P(perm)**	**P(MC)**	**Factor**	**Groups**	**t**	**P(perm)**	**P(MC)**
"Zo"	H, L	5.30	0.0008	**0.0004**	"Tr"	P, C	8.10	0.03	**0.0002**
						P, AC	14.60	0.03	**0.0001**
						C, AC	0.70	0.70	0.46
					"Zo"	H, L	2.69	0.02	**0.02**

June				
**Factor**	**Groups**	**t**	**P(perm)**	**P(MC)**
"TrxZo"	**Term 'TrxZo' for pairs of levels of factor Treatment', within level 'H' of factor 'Zone'**			
	P, C	7.79	0.03	**0.0001**
	P, AC	8.27	0.03	**0.0001**
	C, AC	0.39	0.77	0.69
	**Term 'TrxZo' for pairs of levels of factor 'Treatment', within level 'L' of factor 'Zone'**			
	P, C	6.86	0.02	**0.0007**
	P, AC	6.20	0.02	**0.0009**
	C, AC	0.23	0.85	0.82
	**Term 'TrxZo' for pairs of levels of factor 'Zone', within level 'P' of factor 'Treatment'**			
	H, L	0.98	0.43	0.39
	**Term 'TrxZo' for pairs of levels of factor 'Zone', within level 'C' of factor 'Treatment'**			
	H, L	7.89	0.02	**0.004**
	**Term 'TrxZo' for pairs of levels of factor 'Zone'. within level 'AC' of factor 'Treatment'**			
	H, L	2.42	0.10	0.09

Table S1.2. Pairwise tests on fish herbivory for Pointe de la Cuisse. The pairwise tests were performed on the factor ‘Treatment’ and ‘Zone’ when they were significant. Tr: Treatment, Zo: Zone, H: High Zone, L: Low Zone, P: protected, C: control, AC: artefact control treatment.

March					May					June				
**Factor**	**Groups**	**t**	**P(perm)**	**P(MC)**	**Factor**	**Groups**	**t**	**P(perm)**	**P(MC)**	**Factor**	**Groups**	**t**	**P(perm)**	**P(MC)**
“Zo”	H, L	5.28	0.0009	0.0008	"Tr"	P, C	5.42	0.02	**0.001**	"Tr"	P, C	2.90	0.05	**0.02**
						P, AC	4.30	0.02	**0.005**		P, AC	3.26	0.02	**0.01**
						C, AC	0.23	0.83	0.82		C, AC	0.70	0.48	0.50
					“Zo”	H, L	5.77	0.0004	**0.0005**					

Table S2.1. Pairwise tests on the algal size for Pointe du Rubé. The pairwise tests were performed on the factor ‘Treatment’ and ‘Zone’ when they were significant. Tr: Treatment, Zo: Zone, H: High Zone, L: Low Zone, P: protected, C: control, AC: artefact control treatment.

March					May					June				
**Factor**	**Groups**	**t**	**P(perm)**	**P(MC)**	**Factor**	**Groups**	**t**	**P(perm)**	**P(MC)**	**Factor**	**Groups**	**t**	**P(perm)**	**P(MC)**
“Zo”	H, L	6.5	0.0003	**0.0002**	"Tr"	P, C	12.3	0.02	**0.0001**	"Tr"	P, C	9.09	0.02	**0.0002**
						P, AC	6.6	0.03	**0.0006**		P, AC	9.39	0.02	**0.0002**
						C, AC	2.5	0.05	0.05		C, AC	0.31	0.66	0.76
					“Zo”	H, L	5.04	0.001	**0.0007**	“Zo”	H, L	5.42	0.0007	**0.0007**

Table S2.2. Pairwise tests on the algal size for Pointe de la Cuisse. The pairwise tests were performed on the factor ‘Treatment’ and ‘Zone’ when they were significant. Tr: Treatment, Zo: Zone, H: High Zone, L: Low Zone, P: protected, C: control, AC: artefact control treatment.

March					May					June				
**Factor**	**Groups**	**t**	**P(perm)**	**P(MC)**	**Factor**	**Groups**	**t**	**P(perm)**	**P(MC)**	**Factor**	**Groups**	**t**	**P(perm)**	**P(MC)**
“Zo”	H, L	8.2	0.0002	**0.0001**	"Tr"	P, C	3.53	0.02	**0.011**	"Tr"	P, C	5.33	0.02	**0.0018**
						P, AC	1.43	0.16	0.20		P, AC	5.27	0.03	**0.0021**
						C, AC	1.47	0.29	0.19		C, AC	0.15	0.94	0.88
					“Zo”	H, L	5.31	0.0007	**0.0012**	“Zo”	H, L	9.75	0.0002	**0.0001**

Table S3.1. **Pairwise tests on the biomass for Pointe du Rubé**. The pairwise tests were performed on the interaction 'Tr x Zo' that resulted statistically significant at Pointe du Rubé. Tr: Treatment, Zo: Zone, H: High level, L: Low level, P: protected, C: control, AC: artefact control.

Factor	Groups	t	P(perm)	P(MC)
"TrxZo"	**Term 'TrxZo' for pairs of levels of factor 'Treatment', within level 'H' of factor 'Zone'**			
	P, C	4.82	0.02	**0.002**
	P, AC	47.73	0.03	**0.003**
	C, AC	0.62	0.6	0.54
	**Term 'TrxZo' for pairs of levels of factor 'Treatment', within level 'L' of factor 'Zone'**			
	P, C	53.93	0.03	**0.001**
	P, AC	56.72	0.03	**0.001**
	C, AC	0.24	0.91	0.81
	**Term 'TrxZo' for pairs of levels of factor 'Zone', within level 'P' of factor 'Treatment'**			
	H, L	46.12	0.03	**0.02**
	**Term 'TrxZo' for pairs of levels of factor 'Zone', within level 'C' of factor 'Treatment'**			
	H, L	36.57	0.04	**0.03**
	**Term 'TrxZo' for pairs of levels of factor 'Zone', within level 'AC' of factor 'Treatment'**			
	H, L	54.79	0.03	**0.012**

Table S3.2. Pairwise tests on the biomass for Pointe de la Cuisse. The pairwise tests were performed for the factor 'Treatment' and 'Zone'. Tr: Treatment, Zo: Zone, H: High level, L: Low level, P: protected, C: control, AC: artefact control.

Factor	Groups	t	P(perm)	P(MC)
"Tr"	P, C	3.52	0.02	**0.01**
	P, AC	5.36	0.02	**0.001**
	C, AC	1.17	0.34	0.28

Factor	Groups	t	P(perm)	P(MC)
"Zo"	H, L	3.13	0.01	**0.01**

Table S4.1. Pairwise tests on fertility for Pointe du Rubé. The pairwise tests were performed on the interaction. Tr: Treatment, Zo: Zone, H: High level, L: Low level, P: protected, C: control, AC: artefact control.

Factor	Groups	t	P(perm)	P(MC)
"TrxZo"	**Term 'TrxZo' for pairs of levels of factor 'Treatment', within level 'H' of factor 'Zone'**			
	P, C	2.88	0.02	**0.02**
	P, AC	2.88	0.03	**0.02**
	C, AC	9.21E-2	0.97	0.92
	**Term 'TrxZo' for pairs of levels of factor 'Treatment', within level 'L' of factor 'Zone'**			
	P, C	2.91	0.02	**0.03**
	P, AC	2.82	0.02	**0.02**
	C, AC	1.00	0.34	0.35
	**Term 'TrxZo' for pairs of levels of factor 'Zone', within level 'P' of factor 'Treatment'**			
	H, L	2.73	0.09	0.07
	**Term 'TrxZo' for pairs of levels of factor 'Zone', within level 'C' of factor 'Treatment'**			
	H, L	3.46	0.05	**0.03**
	**Term 'TrxZo' for pairs of levels of factor 'Zone', within level 'CA' of factor 'Treatment'**			
	H, L	1.30	0.26	0.27

Table S4.2. Pairwise tests on fertility for Pointe de la Cuisse. The pairwise tests were performed on the interaction that resulted statistically significant at Pointe de la Cuisse. Tr: Treatment, Zo: Zone, H: High level, L: Low level, P: protected, C: control, AC: artefact control.

Factor	Groups	t	P(perm)	P(MC)
"TrxZo"	**Term 'TrxZo' for pairs of levels of factor Treatment', within level 'H' of factor 'Zone'**			
	P, C	6.53	0.02	**0.0001**
	P, AC	6.75	0.02	**0.0006**
	C, AC	1.74E-02	1	0.98
	**Term 'TrxZo' for pairs of levels of factor 'Treatment', within level 'L' of factor 'Zone'**			
	P, C	3.00	0.02	**0.02**
	P, AC	3.05	0.02	**0.02**
	C, AC	1.88	0.14	0.10
	**Term 'TrxZo' for pairs of levels of factor 'Zone', within level 'P' of factor 'Treatment'**			
	H, L	4.00	0.04	**0.02**
	**Term 'TrxZo' for pairs of levels of factor Zone', within level 'C' of factor 'Treatment'**			
	H, L	1.92	0.17	0.15
	**Term 'TrxZo' for pairs of levels of factor 'Zone', within level 'AC' of factor 'Treatment'**			
	H, L	3.72	0.05	**0.03**

PERMDISP tests. Dispersion tests were performed on each site, sampling time, *Cystoseira* feature and factor of the split-plot design. Significant p-values are reported in bold type.

		March								May								June							
		Pointe du Rubé				Pointe de la Cuisse				Pointe du Rubé				Pointe de la Cuisse				Pointe du Rubé				Pointe de la Cuisse			
		**df1**	**df2**	**F**	***p***	**df1**	**df2**	**F**	***p***	**df1**	**df2**	**F**	***p***	**df1**	**df2**	**F**	***p***	**df1**	**df2**	**F**	***p***	**df1**	**df2**	**F**	***p***
**Number of bites**	**Tr**	2	21	0.1	0.8	2	21	0.3	0.7	2	21	0.8	0.4	2	21	1.2	0.3	2	21	0.7	0.5	2	21	0.2	0.8
	**Zone**	1	22	7.5	**0.01**	1	22	15.1	**0.0008**	1	22	0.02	0.8	1	22	1.2	0.3	1	22	1.7	0.2	1	22	7.1E-04	1
	**Blocks**	3	20	5	**0.09**	3	20	0.8	0.5	3	20	1.3	0.2	3	20	0.2	0.9	3	20	0.6	0.6	3	20	0.9	0.5
**Algal length**	**Tr**	2	21	1	0.4	2	21	0.1	0.9	2	21	2	0.1	2	21	1.2	0.3	2	21	0.6	0.6	2	21	0.2	0.8
	**Zone**	1	22	2.3	0.1	1	22	6.7	**0.01**	1	22	0.4	0.5	1	22	0.3	0.6	1	22	1.3E-05	1	1	22	0.7	0.4
	**Blocks**	3	20	0.7	0.5	3	20	0.1	0.9	3	20	0.5	0.7	3	20	1.4	0.3	3	20	0.1	0.9	3	20	0.2	0.9
**Biomass**	**Tr**																	2	21	6.6	**0.006**	2	21	3	0.07
	**Zone**																	1	22	1.5	0.2	1	22	1.4	0.2
	**Blocks**																	3	20	0.3	0.8	3	20	0.1	1
**Number of receptacles**	**Tr**																	2	21	4.6	**0.02**	2	21	10	**0.0008**
	**Zo**																	1	22	2.2	0.1	1	22	2.3	0.1
	**Blocks**																	3	20	0.5	0.6	3	20	0.1	0.9
